# Memory enhancement produced by post-training exposure to sucrose-conditioned cues

**DOI:** 10.12688/f1000research.2-22.v1

**Published:** 2013-01-23

**Authors:** Matthew R Holahan, Norman M White

**Affiliations:** 1 Department of Neuroscience, Carleton University, 1125 Colonel By Drive, 325 LSRB, Ottawa, ON, K1S 5B6, Canada; 2 Department of Psychology, McGill University, Dr. Penfield Ave, Room N8/3, Montreal, QC, H3A 1B1, Canada

## Abstract

A number of aversive and appetitive unconditioned stimuli (such as shock and food) are known to produce memory enhancement when they occur during the post-training period. Post-training exposure to conditioned aversive stimuli has also been shown to enhance memory consolidation processes. The present study shows for the first time that post-training exposure to conditioned stimuli previously paired with consumption of a sucrose solution also enhances memory consolidation. Male Long Evans rats were trained on a one-session conditioned cue preference (CCP) task on a radial arm maze. Immediately or 2 hours after training, rats consumed a sucrose solution or were exposed to cues previously paired with consumption of sucrose or cues previously paired with water. Twenty-four hours later, the rats were tested for a CCP. Immediate, but not delayed, post-training consumption of sucrose enhanced memory for the CCP. Immediate, but not delayed, post-training exposure to cues previously paired with sucrose, but not with water, also enhanced CCP memory. The possibility that rewarding and aversive conditioned stimuli affect memory by a common physiological process is discussed.

## Introduction

Certain kinds of events that occur during the period immediately after a new task is learned can modulate memory for the task ^1– 5^. Events such as electroconvulsive shock ^6– 8^ or anaesthetization ^9, 10^ can weaken a memory; events such as injections of epinephrine ^11– 13^ or amphetamine ^14– 18^ can strengthen a memory. In all such demonstrations, the post-training treatment was given shortly after a learning trial and retention was measured one or more days later. When administration of the same treatments was delayed for one or more hours after training, they were ineffective. This pattern of effects is consistent with the idea that memory consolidation is a time-limited process ^5, 19^ during which the neural representation of the memory is labile and subject to change ^20– 22^.

The memory enhancing effects of post-training aversive events such as footshock ^23– 25^ and of rewarding events such as electrical self-stimulation of the brain ^26^, and ingestion or injection of sucrose or glucose ^27– 33^ are well-documented. Furthermore, Holahan and White ^34, 35^ found that post-training exposure to conditioned aversive cues previously paired with shock also enhanced memory consolidation. The present study was designed to determine if post-training exposure to rewarding conditioned cues, previously paired with consumption of sucrose, can also enhance consolidation of the memory for a recently acquired task.

## Materials and methods

### Subjects

Subjects were 36 test naïve, male Long-Evans rats (Charles River, St. Constant, Québec, Canada; strain code: 006) that weighed 250–275 g (56–58 days old) at the start of the experiment. They were housed in individual cages with free access to water. The temperature (22°C) and lighting (lights on: 0700 to 1900) of the animal housing unit were controlled. Behavioral testing took place from 1000 to 1400. Care of rats and all procedures were conducted in accordance with the guidelines of the Canadian Council on Animal Care and protocols approved by the McGill University Animal Care Committee (protocol number: 1417) as well as the Guide for the Use and Care of Laboratory Animals.

### Apparatus

***Radial-arm maze.*** The maze was located in a windowless 2.8 × 3.7 × 2.8 m (w × l × h) room partitioned with a sound attenuating divider (1.2 m long) that created a 2.8 × 2.3 m area for the maze. This area contained a number of distal cues. A video camera hung about 1 meter above the maze.

The maze was made of wood painted flat gray and consisted of an octagonal center platform 29.3 cm edge to edge with arms 42.8 cm long and 9 cm wide. Each arm was surrounded by a wall 15.8 cm high at the entrance; 10 cm out from the entrance the wall height decreased to 5 cm. The surface of the maze was 54 cm from the floor of the room. Wooden blocks (30 cm high) also painted flat gray were placed in the entrances of unused arms. Similar blocks with wooden panels attached (28 cm wide) were used to confine a rat to a 35 cm ^2^ area at the end of an arm during training. The panels confined a rat’s view of the room to an arc of approximately 180° facing away from the maze.

***Conditioning boxes.*** This apparatus consisted of a large box made of wood, except the front wall, which was Plexiglas. The box was divided into two compartments of equal size (45 × 45 × 30 cm) by a wooden partition. One of the compartments was painted grey; the other was painted with vertical black and white stripes. The floors in both compartments were made of wood and covered with wood chips. The apparatus was situated in a room across a hallway from the radial arm maze room.

### Procedure

Groups of 7–8 rats were habituated to handling daily for 5 days. They were placed into a large plastic box (70 × 54 × 33 cm) with wood chips covering the floor for 2 h per day while the experimenter picked up and held each rat for 5 min. During these 5 days, food was removed from the rats’ home cages. When returned to its home cage after handling, each rat was given 10 Froot Loops (Kellogg’s, Mississauga, ON) and approximately 5 g of rat chow. At the end of the handling period, all rats weighed 85% ± 3% of their initial free feeding weights. They were maintained at these weights throughout the remainder of the experiment by adjusting the number of pellets given after testing each day. Froot Loops were not given in the home cage after the 5-day handing period.

On days 3–5 of the handling period, water was removed from each rat's cage at 17h00 and replaced with a bottle containing 20 ml of a 10% (w/v water) sucrose solution. At 8h00 the next morning the sucrose bottles were removed and replaced with water.

## Experiment 1: Post-training Sucrose

This experiment compared the effects of immediate and delayed post-training consumption of sucrose on memory for a conditioned cue preference (CCP) ^36^.

### Procedure

Each rat was randomly assigned to a different pair of food-paired and unpaired arms on the radial maze. Food-paired arms contained 30 Froot Loops; unpaired arms were empty. The two arms assigned to each rat were separated by at least 2 other arms. Rats were confined in their food-paired arms for 5 min and then moved to their unpaired arms for 5 min. This sequence was then repeated once more ^35^. Placement on the two arms was always done in this order so that exposure to the unpaired arm was last. When a rat was moved between arms, the experimenter lifted the rat off the arm and placed it onto the other arm. The number of Froot Loops remaining in the paired arm was counted at the end of training and subtracted from 30 to provide a measure of consumption.

Each rat was returned to its home cage when its maze training trials were complete. Rats in the immediate post-training sucrose condition (n=6) were given a 20 ml bottle of 10% sucrose in their home cages and allowed to drink for 20 min. Rats in the delay condition (n=6) were given access to the same solution 2 hrs after being returned to their cages. After the 20 min access period the sucrose bottles were replaced with water bottles for the rats in both groups.

24 hours after the post-training treatments, each rat was placed on the center platform of the maze with the food-paired and unpaired arms open and no food in either arm. The entrances to the other arms were blocked. Each rat was allowed to move freely for 20 min and observed on a monitor connected to the TV camera above the maze. The times of entry into and exit from each arm were recorded and used to calculate the total time spent in each arm. An entry into or an exit from an arm was scored when a rat’s shoulders crossed a line separating the arm from the central platform.

### Results

**Figure 1.**
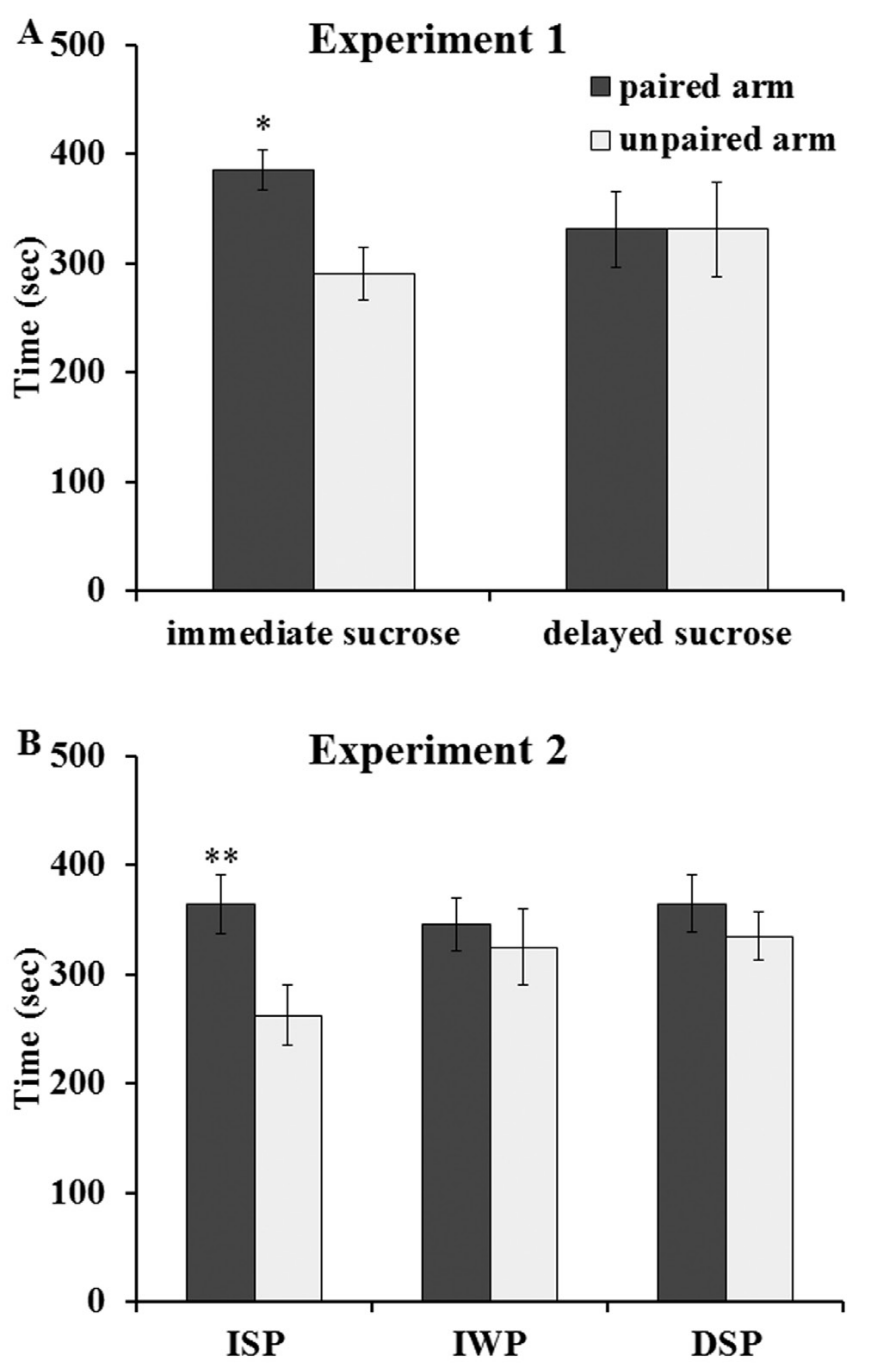
Memory enhancement produced by post-training exposure to sucrose or sucrose-paired cues. **A**) Experiment 1: Modulation by Unconditioned Sucrose. The group exposed to sucrose immediately after training on the radial arm maze conditioned cue preference (CCP) task spent more time in the food-paired arm (* p < 0.05 vs. unpaired arm) 24 hours after training while the group exposed to sucrose 2 hours after CCP training did not spend more time in the food-paired arm compared to the unpaired arm. **B**) Experiment 2: Modulation by Sucrose-Conditioned Cue. The group exposed to sucrose-paired cues immediately after CCP training (group ISP – immediate sucrose paired) spent more time in the food-paired arm 24 hours after training (** p < 0.01 vs. unpaired arm) while neither the group exposed to water-paired cues immediately after training (group IWP – immediate water paired) nor the group exposed to sucrose-paired cues 2 hours after training (group DSP – delayed sucrose paired) spent more time in the food-paired arm. Data are expressed as mean time (in seconds) ± SEM.

The results are shown in Figure 1A. The amounts of time spent in the paired and unpaired arms by the rats in each experimental group were compared using pairwise planned comparisons ^37^, (p. 73) following a two-way ANOVA (group by arm) with one repeated measure. The rats in the immediate post-training sucrose group spent significantly more time in their food-paired than in their unpaired arms (F(1,10) = 5.03, p < 0.05) while the group that drank sucrose 2 hours after maze training spent similar amounts of time in their food-paired and unpaired arms (F(1,10) < 1.0). These results show that immediate, but not delayed; post-training exposure to sucrose had a retroactive enhancing effect on memory consolidation rather than a proactive effect on performance.

Post-training sucrose experiment This experiment compared the effects of immediate and delayed post-training consumption of sucrose on memory for a conditioned cue preference (CCP).

Table 1 shows the mean number of Froot Loops consumed during preference training on the radial maze and the mean volume of sucrose consumed during the post-training period for each of the treatment groups. These data were analyzed with independent samples t-tests. There was no difference in the number of Froot Loops consumed (t(10) < 1.0) but significantly more sucrose was consumed by the 2-hour delay group than by the immediate sucrose group (t(10) = 2.23, p = 0.05). Since the immediate sucrose group exhibited enhanced retention but the delayed group did not, it is unlikely that the difference in sucrose consumption can account for the effects on retention.

**Table 1.** Froot loop and sucrose consumption.

*Exp 1*	Froot loops (mean No ± SEM)	Sucrose (mean mL ± SEM)
Immed Sucrose	14.3 ± 1.3	7.8 ±1.1
Delayed Sucrose	15.0 ± 1.4	13.2 ± 2.1#
***Exp 2***		
Imm Sucrose Paired (ISP)	14.8 ± 0.4	13.8 ± 1.0
Imm Water Paired (IWP)	13.9 ± 0.5	14.5 ± 0.5
Delayed Sucrose Paired (DSP)	12.8 ± 0.6*	13.0 ± 0.9

#p < 0.05 vs. immed sucrose group.

*p < 0.05 vs. immed sucrose paired group (ISP).

## Experiment 2: Post-training Sucrose-Conditioned Cues

This experiment tested the hypothesis that exposure to sucrose conditioned cues would modulate memory in the same way as consumption of sucrose did in Experiment 1.

### Procedure

After the initial handling period, all rats in this experiment were given 3, two-day training trials in the conditioning boxes. On one of the two days of each trial each rat was confined in one of the large compartments for 20 min with the 10% sucrose solution. On the other day, each rat was confined in the other large compartment for 20 min with water. The assignment of sucrose-paired compartments and order of sucrose- and water-pairing were counterbalanced within each experimental group.

24 hours after the end of the conditioning trials the rats were trained on the CCP task as described in Experiment 1.

After each rat completed maze training, it was placed in the conditioning box for 20 min. Neither the sucrose solution nor water was available in either compartment. Rats in the immediate sucrose-paired (ISP) condition (n=8) were placed in their sucrose-paired compartments immediately after training; rats in the immediate water-paired condition (IWP; n=8) were placed into their water-paired compartments immediately after training. The rats in the 2-hour delayed sucrose-paired (DSP) condition (n=8) were returned to their home cages for 2 hours with no food or water and were then placed into their sucrose-paired compartments.

24 hours after the post-training treatments all rats were tested on the maze as described in Experiment 1.

### Results

The results are shown in Figure 1B. The rats in the ISP group spent significantly more time in their food-paired than in their unpaired arms (F(1,21) = 9.79, p < 0.01). There was no significant difference in the times spent in the 2 arms by the rats in the IWP group (F(1,21) < 1.0) or for the rats in the DSP group (F(1,21) < 1.0). These results show that immediate, but not delayed, post-training exposure to sucrose-conditioned cues, but not water-conditioned cues, had a retroactive enhancing effect on memory consolidation.

Post-training sucrose-conditioned cues experiment This experiment tested the hypothesis that exposure to sucrose conditioned cues would modulate memory in the same way as consumption of sucrose did in Experiment 1.

Table 1 shows the mean number of Froot Loops consumed on the maze and the average amount of sucrose consumed over the 3 sucrose days. There was a significant difference in the mean number of Froot Loops consumed during preference training on the maze by the rats in the 3 groups (F(2,21) = 3.68, p < 0.05). Tukey's post-hoc tests revealed that the rats given immediate post-training exposure to their sucrose conditioned cues (ISP) ate more Froot-Loops than the rats in the 2 hr delay group (DSP; p < 0.05). It is therefore possible, although unlikely, that the difference in CCP performance of these groups was due to this difference in Froot-Loop consumption. The mean volume of sucrose consumed over the six-day training period in the conditioning box by the rats in each of the treatment groups was analyzed with two-way, repeated measures ANOVA (group by day). This analysis revealed a main effect of day (F(2,4) = 56.53, p < 0.001) indicating all 3 groups increased their sucrose consumption similarly over training days.

## Discussion

Exposure to sucrose or sucrose-conditioned cues immediately after training enhanced retention of information required for expression of a CCP in the radial maze. Increasing the delay between maze training and either post-training treatment to 2 hours eliminated this effect, showing that this is a classic time-dependent memory modulation effect. This appears to be the first demonstration that post-training exposure to appetitive conditioned cues enhances memory consolidation.

The enhanced CCP in the immediate groups was not due to learning about the temporal relationship between exposure to the food-paired of unpaired maze arms and any rewarding or conditioned rewarding effects either of the post-training treatments may have had. This is because the rats were always exposed to their unpaired arms immediately before the treatments. Learning that exposure to an arm led to reward would therefore have increased the time spent in the unpaired arm at the expense of time in the food-paired arm. The fact that the rats in the sucrose and ISP groups spent more time on their food-paired than on their unpaired arms shows that this form of learning did not influence the rats' behavior. Moreover, the absence of modulation in the 2 hour delay groups shows that the enhanced CCP for the food-paired arm was due to a retroactive enhancement of memory.

Immediate post-training exposure to water-paired cues in the IWP group did not have a memory modulation effect. Messier and White ^27^ found that post-training consumption of water failed to modulate memory for a conditioned emotional response in an aversive conditioning situation. If it is true that, unlike consumption of sucrose solutions, consumption of water does not have memory modulating effects, it is not surprising that exposure to conditioned stimuli that have been paired with water also lacks these effects.

A number of other aversive and appetitive unconditioned stimuli (UCS) are known to produce memory modulation when they occur during the post-training period and each of these is also associated with several well-studied unconditioned responses (UCRs). For example, food (the classic UCS) improves retention when consumed during the post-training period ^38^, as does the consumption of sucrose solutions ^27, 28^. Both of these UCSs produce UCRs in the form of salivation ^39^ and glucose or insulin release ^40, 41^, among other responses. Aversive events such as foot shock also modulate memory ^23, 24, 34^ and produce UCRs, including increased heart rate ^42^ and release of stress-related hormones ^29, 43^. When a rat is exposed to a UCS, memory modulation can be seen as resulting from the action of a UCR, either one of those mentioned here, or some other as yet unidentified UCR. These UCRs are subject to conditioning ^44, 45^ so it can be presumed that when they occur as conditioned responses (CRs) after conditioning, they have the same effect as the UCRs.

Interestingly, rewarding and aversive UCSs produce many of the same UCRs and CRs. These include increases in blood glucose ^29, 40, 41, 43^, elevation in catecholamine levels such as norepinephrine and dopamine ^46– 50^ and activation of the hypothalamic-pituitary-adrenal (HPA) axis ^51– 53^, all of which have been shown to modulate memory (see ^4, 54, 55^). Thus, memory modulation by unconditioned rewarding or aversive events and by rewarding or aversive conditioned stimuli may result from similar internal responses.
