# Supplementary material for: Memory enhancement produced by post-training exposure to sucrose-conditioned cues
Source: F1000Res. 2013 Jan 23;2:22. [Version 1] doi: 10.12688/f1000research.2-22.v1 (PMC3790601; doi:10.12688/f1000research.2-22.v1)
Supplement: Peer review history

# Peer review: Referee response for version 1

**Published:** 2013-01-28

**Authors:** Edvard I Moser

**Affiliations:** 1 Centre for the Biology of Memory, Norwegian University of Science and Technology, Trondheim, Norway

I have read this submission. I believe that I have an appropriate level of expertise to confirm that it is of an acceptable scientific standard.

# Peer review: Referee response for version 1

**Published:** 2013-01-25

**Authors:** Kent Berridge

**Affiliations:** 1 Department of Psychology, University of Michigan, Ann Arbor, MI, USA

I have read this submission. I believe that I have an appropriate level of expertise to confirm that it is of an acceptable scientific standard.
